# Binding investigation and preliminary optimisation of the 3-amino-1,2,4-triazin-5(2*H*)-one core for the development of new Fyn inhibitors

**DOI:** 10.1080/14756366.2018.1469017

**Published:** 2018-05-11

**Authors:** Giulio Poli, Margherita Lapillo, Carlotta Granchi, Jessica Caciolla, Nayla Mouawad, Isabella Caligiuri, Flavio Rizzolio, Thierry Langer, Filippo Minutolo, Tiziano Tuccinardi

**Affiliations:** a Department of Pharmacy, University of Pisa, Pisa, Italy;; b Pathology Unit, Department of Molecular Biology and Translational Research, National Cancer Institute and Center for Molecular Biomedicine, Aviano (PN), Italy;; c Department of Molecular Science and Nanosystems, Ca' Foscari Università di Venezia, Venezia-Mestre, Italy;; d Department of Pharmaceutical Chemistry, Faculty of Life Sciences, University of Vienna, Vienna, Austria;; e Sbarro Institute for Cancer Research and Molecular Medicine, Center for Biotechnology, College of Science and Technology, Temple University, Philadelphia, PA, USA

**Keywords:** Fyn kinase, molecular modelling, kinase inhibitors, drug design

## Abstract

Fyn tyrosine kinase inhibitors are considered potential therapeutic agents for a variety of human cancers. Furthermore, the involvement of Fyn kinase in signalling pathways that lead to severe pathologies, such as Alzheimer’s and Parkinson’s diseases, has also been demonstrated. In this study, starting from 3-(benzo[d][1,3]dioxol-5-ylamino)-6-methyl-1,2,4-triazin-5(2*H*)-one (**VS6**), a hit compound that showed a micromolar inhibition of Fyn (IC_50_ = 4.8 μM), we computationally investigated the binding interactions of the 3-amino-1,2,4-triazin-5(2*H*)-one scaffold and started a preliminary hit to lead optimisation. This analysis led us to confirm the hypothesised binding mode of **VS6** and to identify a new derivative that is about 6-fold more active than **VS6** (compound **3**, IC_50_ = 0.76 μM).

## Introduction

Fyn kinase is a member of the Src family kinases (SFKs), which is one of the largest and most studied families of non-receptor tyrosine kinases (TKs) due to the implications of its members in oncogenesis and cancer development. Fyn kinase is involved in a plethora of physiological processes ranging from cell growth, adhesion and motility to ion channels and platelet activation, as well as cytokine receptor and growth factor signaling^1^. At the level of the central nervous system (CNS), Fyn exerts several different functions connected to brain development. In fact, Fyn is involved in axon–glial signal transduction, oligodendrocyte maturation and myelination^2^; it also stimulates the synthesis of abundant myelin associated oligodendrocytic basic protein, thus influencing oligodendroglial morphological differentiation^3^, and it is implicated in synapse formation and post-synaptic excitatory transmission^4^. Moreover, Fyn was found to be implied in T-cell development, homoeostasis, activation and to have a critical role in thymocyte development together with Lck kinase, which is another member of the SKFs^5^. Due to its several physiological roles, an aberrant expression of Fyn kinase or a dysregulation of its activity is involved in the development and progression of different pathological conditions. In fact, Fyn overexpression has been connected to the pathogenesis of various types of tumors^1^, particularly prostate^6^, breast^7^ and ovarian cancer^8^, as well as haematological tumours like chronic myeloid leukemia^9^ and other types of cancer like glioblastoma and neuroblastoma^10^. Moreover, recent studies highlighted the role of Fyn in the resistance of some tumour cells to anti-cancer treatments, such as in tamoxifen-resistant breast cancer cell lines^11^. Recently, Fyn kinase gained more and more attention as a new therapeutic target for the treatment of neurodegenerative pathologies. Fyn plays a key role in the development and progression of Alzheimer’s disease (AD), being involved in the synaptic toxicity and cognitive impairments produced by amyloid-β (Aβ) oligomers and promoting the formation of neurofibrillary tangles by phosphorylating Tau protein^12^. In fact, Fyn kinase, Aβ and Tau protein have been even referred to as the “toxic triad” of AD. Furthermore, Fyn showed to be a mediator of the microglial neuroinflammatory processes typical of Parkinson’s disease (PD) and could thus represent a potential target for the treatment of neurodegenerative diseases that, like PD, are associated with proinflammatory processes involving microglia. Due to its involvement in cancer and CNS pathologies, the identification of selective Fyn inhibitors is an expanding field of study. Several TKs small-molecule inhibitors with activity towards Fyn kinase have been reported in literature^13^. However, potent Fyn inhibitors with high selectivity over other TKs and, in particular, over the other SFKs members are still missing. As an example, the well-known pyrazolo[3,4-*d*]pyrimidine **PP2**, is a low nanomolar inhibitor of Fyn kinase identified by Hanke and collaborators in 1996^14^, which is still commonly used to study the complex network of physiological processes involving Fyn kinase activity^15–17^. Nevertheless, **PP2** is also endowed with a comparable inhibitory activity towards other SFKs, in particular Src and Lck, and demonstrated to be remarkably potent also on other kinases such as CK1δ (Casein kinase 1 δ), RIP2 (receptor-interacting protein kinase 2) and GAK (cyclin G-associated kinase)^18^. Virtual screening (VS) approaches have shown to be a powerful tool for the discovery of new Fyn kinase and SFKs inhibitors^19^. In particular, we recently developed a VS study mixing ligand-based and receptor-based approaches, which allowed the identification of a novel small-molecule inhibitor of Fyn kinase (compound **VS6**) with low micromolar potency^20^. Here, we report the computational investigation of **VS6** binding mode into Fyn catalytic site, carried out through consensus docking, MD simulations and binding free energy evaluations, followed by a preliminary structural optimisation aimed at the development of novel potent Fyn inhibitors.

## Materials and methods

### Molecular modelling

#### Consensus docking studies

The ligands were built by means of Maestro^21^ and were then minimised in a water environment (using the generalised Born/surface area model) by means of Macromodel^22^. The minimisation was performed using the conjugate gradient (CG), the MMFFs force field, and a distance-dependent dielectric constant of 1.0 until the ligands reached a convergence value of 0.05 kcal Å^−1 ^mol^−1^. Nine different docking procedures were applied and for each docking calculation only the best scored pose was taken into account^23–25^. The docking calculations were carried out by using AUTODOCK 4.2.3^26^, DOCK 6.7^27^, FRED 3.0^28–31^, GOLD 5.1 (with the ASP, CSCORE and GSCORE scoring functions)^32^, GLIDE 5.0 (with the SP scoring function)^33^, AUTODOCK VINA 1.1^34^, and PLANTS^35^ accordingly employing the procedures previously described^24^^,^^36^. The ligands were docked into the binding site of human Fyn (2DQ7^37^ PDB code) by using the different docking procedures, then the root mean square deviation (RMSD) of each of these docking poses against the remaining docking results was evaluated using the rms_analysis software of the GOLD suite. The most populated cluster of solutions was then considered and subjected to molecular dynamic (MD) simulations.

##### MD simulations

All simulations were performed using AMBER, version 14^38^. MD simulations were carried out using the ff14SB force field at 300 K in a rectangular parallelepiped water box. The TIP3P explicit solvent model for water was used. Chlorine ions were added as counter ions to neutralise the system. Prior to MD simulations, two steps of minimisation were carried out using the same procedure described above. Particle mesh Ewald (PME) electrostatics and periodic boundary conditions were used in the simulation^39^. The time step of the simulations was 2.0 fs with a cut-off of 10 Å for the non-bonded interactions, and SHAKE was employed to keep all bonds involving hydrogen atoms rigid. Constant volume periodic boundary MD was carried out for 0.5 ns, during which the temperature was raised from 0 to 300 K. Then, 99.5 ns of constant pressure periodic boundary MD was carried out at 300 K using the Langevin thermostat to maintain constant the temperature of our system. All the α carbons of the protein were blocked with a harmonic force constant of 10 kcal/mol·Å^2^. General Amber force field (GAFF) parameters were assigned to the ligands, while partial charges were calculated using the AM1-BCC method as implemented in the Antechamber suite of AMBER 14.

#### Binding energy evaluation

The evaluation of the binding energy associated with the ligand–protein complexes analysed through MD simulations was carried out using AMBER 14. The trajectories relative to the last 50 ns of each simulation were extracted and used for the calculation, for a total of 50 snapshots (at time intervals of 1 ns). Van der Waals, electrostatic and internal interactions were calculated with the SANDER module of AMBER 14, whereas polar energies were calculated using both the Generalised Born and the Poisson − Boltzmann methods with the MM-PBSA module of AMBER 14. Dielectric constants of 1 and 80 were used to represent the gas and water phases, respectively, while the MOLSURF program was employed to estimate the non-polar energies.

##### Fyn kinase assays

Compound **1** was synthesised, compounds **2** and **3** were purchased from Vitas-M Laboratory, whereas **PP2** was purchased from Tocris Bioscience. Full-length recombinant human Fyn, Poly(Glu4, Tyr1) synthetic peptide substrate, ATP stock solution and Kinase-Glo^®^ Luminescent Kinase Assay were purchased from Promega. The kinase reaction was conducted at room temperature at a final volume of 50 μL in 40 mM Tris buffer, pH 7.5, containing 20 mM MgCl_2_ and 0.05 mM DTT. A total of 10 μL of ATP 20 μM were added to 10 μL of Poly(Glu4, Tyr1) synthetic peptide substrate 1 μg/μL and 5 μL of DMSO containing the appropriate amount of compound. The reaction was initiated by the addition of 25 μL of Fyn (36–48 ng/well) in such a way that the assay was linear over 60 min. The final concentration of the analysed compounds ranged from 10 to 0.032 μM for **PP2** and from 200 to 0.01 μM for compounds **1**–**3**. After the reaction had proceeded for 60 min, 50 μL Kinase-Glo^®^ reagent was added to terminate the reaction. This solution was then allowed to proceed for additional 10 min to maximise the luminescence reaction. Values were then measured by using a VictorX3 PerkinElmer instrument for luminosity measurements. Two reactions were also run: one reaction containing no compounds and the second one containing neither inhibitor nor enzyme.

##### Cell viability assay

MDA-MB-231 (human breast carcinoma cells) and A549 (non-small cell lung cancer) were purchased from ATCC and maintained at 37 °C in a humidified atmosphere containing 5% CO_2_ accordingly to the supplier. Cells (10^3^) were plated in a 96-well culture plates. The day after seeding, vehicle or compounds were added at different concentrations to the medium. Compounds were added to the cell culture at a concentration ranging from 200 to 0.1 µM. Cell viability was measured after 96 h according to the supplier (Promega, G7571) with a Tecan M1000 instrument. IC_50_ values were calculated from logistical dose response curves. Mean and standard deviation were reported (*n* = 3).

## Results and discussion

**Figure 1.**
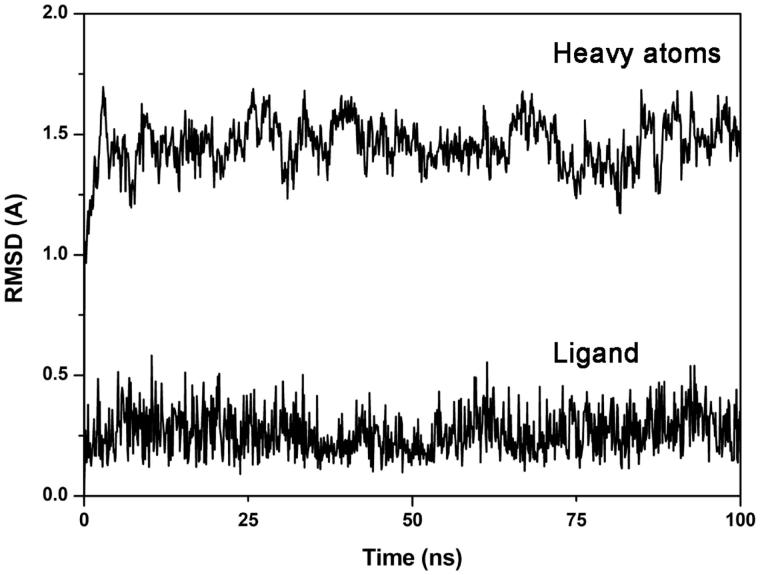
Analysis of the MD simulation of Fyn kinase in complex with **VS6**. The RMSD of the ligand and the heavy atoms of the receptor from the starting model structure during the simulation is reported.

Recently, through a virtual screening (VS) study combining a FLAP ligand-based similarity analysis with docking and MD simulations, we identified few hit compounds endowed with low micromolar Fyn inhibitory potency^20^. Among these, compound **VS6**, (Table 1) bearing a 3-amino-1,2,4-triazin-5(2*H*)-one scaffold, showed the most interesting activity with an IC_50_ value for Fyn inhibition of 4.8 μM. With the aim of paving the way for the structural optimisation of the ligand and for the development of derivatives with higher affinity for Fyn kinase, consensus docking, MD simulations and relative binding free energy evaluations were used to assess the reliability of the binding mode predicted for compound **VS6** within Fyn catalytic site. As a first step of our analysis, compound **VS6** was subjected to our recently developed consensus docking approach combining different docking methods (see Materials and Methods for details), which was found to predict ligand binding poses better than the single docking procedures and to provide hints about binding pose reliability^23^^,^^24^. The consensus docking approach gave a first confirmation of the reliability of the binding disposition into Fyn kinase domain previously proposed for **VS6**, since 6 out of the 9 docking methods tested predicted the same result. The binding mode of compound **VS6** has been further analysed by subjecting this ligand–protein complex to 100 ns of MD simulation. By analysing the root-mean square deviation (RMSD) of all the heavy atoms from the X-ray structures, we observed an initial increase followed by a stabilisation of the RMSD value around 1.5 Å (Figure 1). Regarding the geometry of the ligand, we analysed the trend of the RMSD of its position over time during the simulation with respect to the starting docking pose, and it showed an average RMSD value of about 0.3 Å.

**Table 1.** H-bonds analysis of **VS6** with Fyn during the MD simulation.


acceptor	donor H	donor	% occupied
E343@O	LIG@H	LIG@N5	96.7
LIG@N4	M345@H	M345@N	92.1
T342@OG1	LIG@H	LIG@N8	49.8
LIG@O12	K299@H	K299@NZ	21.6

**Figure 2.**
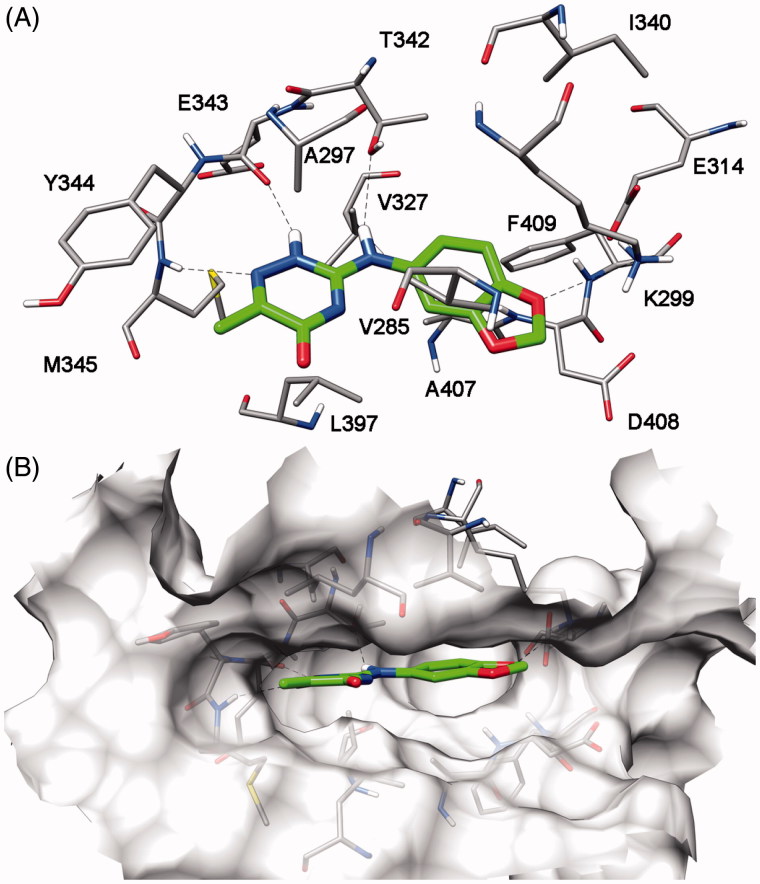
Putative binding mode of compound **VS6** into Fyn kinase. (A) View of the most relevant ligand – receptor interactions and (B) binding pose of the ligand (green) in the binding site.

Figure 2 shows the suggested binding disposition of compound **VS6** refined through MD simulation. The triazinone central core of the compound interacts with the hinge region of the kinase forming two H-bonds with the nitrogen backbone of M345 and the oxygen backbone of E343. Concerning the substituents connected with this scaffold, the 6-methyl group does not show any important interaction and, as shown in Figure 2(B), it is directed outside the binding site towards the solvent exposed region of the protein; the amine group forms an H-bond with the hydroxyl group of T342, and the benzodioxole ring shows lipophilic interactions with V285 and A407 and it is positioned near the positive nitrogen of K299.

As shown in Table 1, the H-bond analysis of the MD simulation confirms the stability of the interactions with the backbone of E343 and M345 since during the whole simulation they are highly conserved (92–97% occupied populations). Differently from these two interactions, the H-bonds with T342 and K299 are maintained only for 50–22% of the whole simulation.

**Table 2.** Structure and Fyn inhibitory activity of the tested compounds.

As a further step of this research study, we were aimed at experimentally verifying the reliability of the proposed binding disposition. From a chemical point of view, the easiest way to confirm this binding mode was the addition of a large substituent in the place of the methyl group in position 6 of the triazinone. As reported above, this methyl group is directed outside the binding site towards the solvent exposed surface of the protein; therefore, a substitution in this point of the molecule should not determine important variations of the compound inhibitory activity. Based on this hypothesis, compound **1**, characterised by the presence of a *p*-fluorobenzyl fragment in position 6 of the triazinone ring, was synthesised (see Supplementary Material) and tested for its inhibitory activity. As shown in Table 2, this compound displayed an IC_50_ value very similar to that possessed by **VS6**, thus supporting the proposed binding hypothesis.

After having obtained the additional experimental support of the proposed **VS6** binding mode, we investigated the role of the aminobenzodioxole fragment. The MD simulation suggested that the amino group was able to interact with the hydroxyl fragment of T342; however, this interaction was not highly stable. A deep analysis of the simulation highlighted that the distance between the oxygen of T342 and the nitrogen of the amine was usually in the range values of an H-bond interaction. However, the conjugation of the amine group with the aromatic benzodioxole and the triazinone ring hindered the rotation of the torsions associated with the nitrogen, leading to a planar configuration of the benzodioxolylaminotriazinone. With this geometry, the amine moiety was not able to efficiently interact with the oxygen of T342, due to the donor – acceptor H-bond angle that showed an average value of about 140°. Concerning the benzodioxole ring, the MD simulation analysis shows lipophilic interactions and also an additional H-bond contribution for the interaction of this compound with K299. In order to evaluate the role of the T342 and K299 H-bonds with the ligand, the benzodioxole ring was replaced with a phenyl (compound **2**) and a cyclohexyl ring (compound **3**). These two compounds, which were not previously tested as inhibitors of Fyn or other kinases, were thus docked into the Fyn-binding site and then subjected to the MD simulation protocol applied for compound **VS6**. Table 3 shows the H-bond analysis of the MD simulation for the two compounds; obviously, for both compounds there were no H-bond interactions with K299. Furthermore, in agreement with the planar geometry of the amine group of the ligand, compound **2** showed an H-bond interaction scheme similar to that showed by **VS6**, with two highly stable interactions with the backbone of E343 and M345 (91–100% occupied populations) and a less stable interaction with T342 (48% occupied population). The replacement of the aromatic ring with the cyclohexyl group should allow a level of amine torsional freedom greater than that possessed by **VS6**. Interestingly, as shown in Table 3, all the three H-bond interactions formed by compound **3** were highly stable with occupancy values higher than 86%.

**Table 3.** H-Bonds analysis of compounds **2** and **3** with Fyn during the MD simulation.


acceptor	donor H	donor	% occupied
**Compound 2**			
E343@O	LIG@H	LIG@N5	99.6
LIG@N4	M345@H	M345@N	91.4
T342@OG1	LIG@H	LIG@N8	47.6
**Compound 3**			
E343@O	LIG@H	LIG@N5	99.9
LIG@N4	M345@H	M345@N	86.8
T342@OG1	LIG@H	LIG@N8	91.0

The three MD trajectories of compounds **VS6** and **2**–**3** were further analysed through the Molecular Mechanics-Poisson–Boltzmann Surface Area (MMPBSA) method^40^ that has been shown to accurately estimate the ligand–receptor energy interaction^41–45^. This approach averages the contributions of gas-phase energies, solvation free energies and solute entropies calculated for snapshots of the complex molecule as well as the unbound components, extracted from MD trajectories, according to the procedure fully described in Materials and Methods. As shown in Table 4, this analysis highlighted that compound **3** should be more active than the other two compounds, mainly due to an increase of the electrostatic contribution that could be due to the stable interaction with T342, which compensates the absence of the secondary interaction with K299. Conversely, compound **2** was predicted to be less potent than reference compound **VS6**, probably because it showed an interaction with T342 similar to that observed for **VS6** and the loss of the secondary interaction with K299.

**Table 4.** MM-PBSA results for the complexes of Fyn with **VS6** and compounds **2**–**3**. ΔPBSA is the sum of the electrostatic (Ele), van der Waals (VdW), polar (PB) and non-polar (PBSur) solvation free energy. Data are expressed as kcal•mol^−1^.

	Ele	VdW	PBsur	PB	ΔPBSA
**VS6**	−27.5	−35.0	−3.1	36.5	−29.1
**2**	−26.5	−32.9	−2.8	34.9	−27.3
**3**	−28.5	−34.4	−3.0	33.5	−32.4

Compounds **2** and **3** were commercially available and were thus purchased and tested for their Fyn inhibitory activity. Interestingly, in agreement with the computational studies, compound **2** showed an IC_50_ higher than 100 µM, whereas compound **3** displayed a nearly sixfold increase of activity with respect to **VS6** with an IC_50_ in the submicromolar range. In order to compare the binding mode predicted for compound **3** with respect to **PP2**, the reference inhibitor was docked into Fyn catalytic site by applying the same computational procedure used for the other compounds reported in this paper. As shown in Figure S3 in the Supplementary Material, the aminopirimidine ring of **PP2** well matches the triazinone core of compound **3** and the two ligands share the H-bonds with the backbone of E343 and M345. On the contrary, the aniline moiety of **PP2** is not able to assume a disposition allowing the formation of the H-bond with the side chain of T342 predicted for compound **3**. Nevertheless, the *p*-chlorophenyl ring of **PP2** perfectly fills the predominantly hydrophobic pocket constituted by M318, I340, T342 and the side chains of K299 and E314, thus forming several additional lipophilic interactions with respect to the cyclohexyl ring of compound **3**. Moreover, the tert-butyl group of **PP2** forms further hydrophobic interactions with L277, V285 and L397 that are not observed for compound **3**.

Compound **3** was further tested in *in vitro* experiments to evaluate its antiproliferative potencies on cancer cells and **PP2** was used as a reference compound. To this aim, two tumour cell lines were chosen, the human non-small-cell lung A549 and the highly invasive human breast MDA-MB-231 cancer cells, due to the critical role that Fyn plays in the tumour progression and development of metastases in these two types of tumors^46^^,^^47^. Overall, compound **3** produced an appreciable inhibition of cell viability, with IC_50_ values ranging from 35 to 101 μM (Table 5) that were lower than those showed by **VS6** (IC_50_ = 145.0 and 198.2 for A549 and MDA-MB-231, respectively)^20^.

**Table 5.** Cell growth inhibitory activities (IC_50_) of compounds **3** and **PP2**.

Cancer cell line	Tissue of origin	IC_50_ values (µM)	
		3	PP2
A549	Lung	101.0 ± 10.8	14.3 ± 2.0
MDA-MB-231	Breast	34.8 ± 4.6	12.0 ± 1.4

In conclusion, according to our consensus docking and MD simulation results, we can confirm the reliability of the binding mode predicted for compound **VS6** within Fyn catalytic site. In addition, the replacement of the benzodioxole moiety with a cyclohexyl ring led to compound **3** endowed with a six-fold increased activity and highlighted the role of the H-bond between the ligand and residue T342. Finally, compound **3**, tested on human cancer cell lines, proved to inhibit cell proliferation with an appreciable antiproliferative activity (IC_50_ from 35 to 101 μM).

## Supplementary Material

IENZ_1469017_Supplementary_Material.pdf
