# Supplementary material for: Binding investigation and preliminary optimisation of the 3-amino-1,2,4-triazin-5(2H)-one core for the development of new Fyn inhibitors
Source: J Enzyme Inhib Med Chem. 2018 May 11;33(1):956–61. doi: 10.1080/14756366.2018.1469017 (PMC6009924; doi:10.1080/14756366.2018.1469017)
Supplement: IENZ_1469017_Supplementary_Material.pdf [file IENZ_A_1469017_SM3837.pdf]

Binding investigation and preliminary optimization of the 3-amino-1,2,4-triazin-5(2*H*)-one core for the development of new Fyn inhibitors

Giulio Poli,[1] Margherita Lapillo,[1] Carlotta Granchi,[1] Jessica Caciolla,[1] Nayla Mouawad,[1,2] Isabella Caligiuri,[2] Flavio Rizzolio,[3] Thierry Langer,[4] Filippo Minutolo,[1] Tiziano Tuccinardi[1,5]

[1]Department of Pharmacy, University of Pisa, Pisa, Italy, [2]Pathology Unit, Department of Molecular Biology and Translational Research, National Cancer Institute and Center for Molecular Biomedicine, Aviano (PN), Italy, [3]Department of Molecular Science and Nanosystems, Ca' Foscari Università di Venezia, Venezia-Mestre, Italy, [4]Department of Pharmaceutical Chemistry, Faculty of Life Sciences, University of Vienna, Vienna, Austria, [5]Sbarro Institute for Cancer Research and Molecular Medicine, Center for Biotechnology, College of Science and Technology, Temple University, Philadelphia, PA, USA

Address for correspondence: Tiziano Tuccinardi, Department of Pharmacy, University of Pisa, Via Bonanno 6, 56126 Pisa, Italy. E-mail: tiziano.tuccinardi@unipi.it

Table of Content

Chemistry — Pags 2-5

^{1}H-NMR spectrum of intermediate **5** — Pag 7

HPLC report of final compound **1** — Pag 8

Superimposition between the interaction of compound **3** and PP2 with Fyn — Pag 9

Materials and methods.

CHEMISTRY

General Procedures and Materials. All solvents and chemicals were used as purchased without further purification from Aldrich-Merck or Alfa Aesar-Thermo Fisher. Compounds **2** and **3** were purchased from Vitas-M Laboratory and used without further purification. Chromatographic separations were performed on silica gel columns by flash chromatography (Kieselgel 40, 0.040−0.063 mm; Merck). Reactions were followed by thin layer chromatography (TLC) on aluminum silica gel (60 F254) sheets that were visualized under a UV lamp. Evaporation was performed in vacuo (rotating evaporator). Sodium sulfate was always used as the drying agent. Proton (^{1}H) and carbon (^{13}C) NMR spectra were obtained with a Bruker Avance III 400 MHz spectrometer using the indicated deuterated solvents. Chemical shifts are given in parts per million (ppm) (δ relative to residual solvent peak for ^{1}H and ^{13}C). Yields refer to isolated and purified products derived from non optimized procedures. HPLC analysis: compound **1** is $\geq$ 95% pure by HPLC, confirmed via UV detection (λ = 254 nm). Analytical reversed-phase HPLC was conducted using a Kinetex EVO C18 column (5 µm, 150 mm × 4.6 mm, Phenomenex, Inc.); eluent A, water; eluent B, CH_3CN; after 5 min at 25% B, a gradient was formed from 25% to 75% of B in 5 min and held at 75% of B for 10 min; flow rate was 1 mL/min.

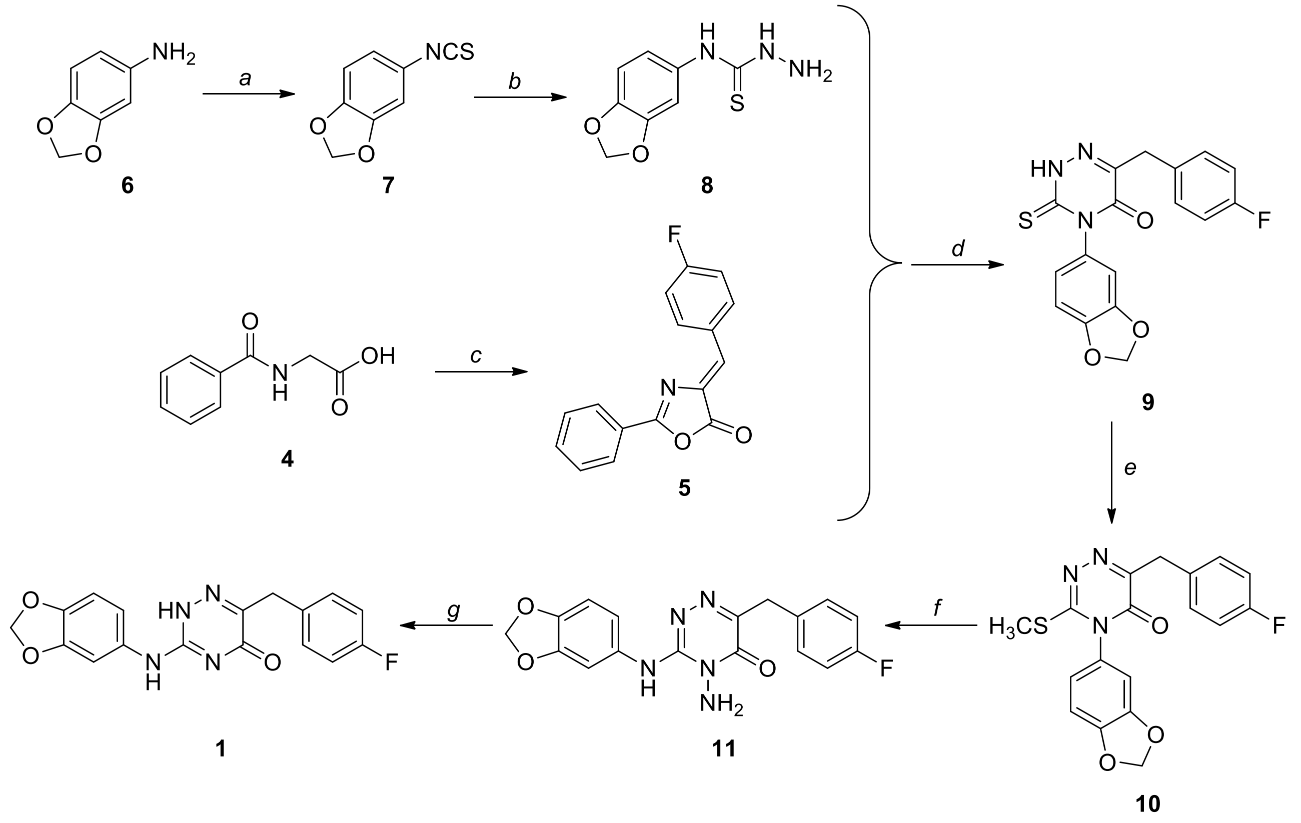

Scheme 1. *Reagents and conditions*: a) CS_2, Et_3N, EtOH, rt; then Boc_2O, DMAP, EtOH, rt; b) NH_2NH_2 50-60%, *i*PrOH, rt; c) 4-fluorobenzaldehyde, Ac_2O, AcOK, 90 °C; d) 1M KOH, EtOH, reflux; e) 25% NaOMe/MeOH, MeOH, MeI, 0 °C then rt; f) NH_2NH_2 50-60%, *i*PrOH, reflux; g) aq. 10% $NaNO_2$, conc. HCl, EtOH, rt.

5-Isothiocyanatobenzo[*d*][1,3]dioxole (**7**). A solution of 3,4-(methylenedioxy)aniline **6** (500 mg, 3.64 mmol) in EtOH (7.3 mL) was treated with carbon disulfide (0.66 mL, 11 mmol) followed by triethylamine (0.51 mL, 3.6 mmol). After stirring at room temperature for 30 min., a solution of Boc_2O (794 mg, 3.64 mmol) in EtOH (3.6 mL) and DMAP (13.3 mg, 0.109 mmol) were added to the reaction mixture at 0 °C. The reaction was stirred at room temperature for 20 min. After the reaction was complete, the solvent was evaporated and the crude was purified by silica gel column

chromatography (*n*-hexane/EtOAc 98:2) to afford compound **7** as a white solid (481 mg, 74 % yield). ^{1}H-NMR (DMSO-d_6) δ ppm: 6.94-6.97 (m, 2H), 7.12 (t, 1H, J = 1.2 Hz).

N-(Benzo[*d*][1,3]dioxol-5-yl)hydrazinecarbothioamide (**8**). To a stirred solution of hydrazine hydrate (50-60%, 80 µL, 2.7 mmol) in 22.8 mL of isopropanol, compound **7** (481 mg, 2.28 mmol) was added at room temperature. Precipitate was formed immediately. Stirring was continued for 1 h. Then the mixture was filtered and the precipitate was washed with isopropanol and dried under vacuum to give derivative **8** as a white solid (480 mg, 99 % yield), which was used in the next step without further purification. ^{1}H-NMR (DMSO-d_6) δ ppm: 4.72 (bs, 2H), 6.83 (d, 1H, J = 8.3 Hz), 6.86-6.95 (bm, 1H), 7.24-7.34 (bm, 1H), 9.03 (s, 1H), 9.50 (bs, 1H).

(*Z*)-4-(4-Fluorobenzylidene)-2-phenyloxazol-5(*4H*)-one (**5**).[1,2] A mixture of 4-fluorobenzaldehyde (0.43 mL, 4.0 mmol), hippuric acid **4** (722 mg, 4.03 mmol) and potassium acetate (396 mg, 4.03 mmol) in acetic anhydride (1.1 mL, 12 mmol) was stirred at 90 °C for 2 h in a sealed vial and then warmed slowly to room temperature. The reaction was quenched with water and extracted with EtOAc. The organic layer was washed sequentially with water and brine, dried over Na_2SO_4 and the solvent was removed under reduced pressure. The solid residue was repeatedly washed with *n*-hexane and then dried under vacuum to obtain the desired product **5** as a yellow solid (793 mg, 74 % yield). ^{1}H-NMR ($CDCl_3$) δ ppm: 7.18 (double AA'XX', 2H, $^{3}J_{HF-o}$ = 9.7 Hz, J_{AX} = 8.7 Hz, $J_{AA'/XX'}$ = 2.4 Hz), 7.22 (s, 1H), 7.51-7.58 (m, 2H), 7.63 (tt, 1H, J = 7.4, 1.6 Hz), 8.16-8.21 (m, 2H), 8.24 (double AA'XX', 2H, $^{4}J_{HF-m}$ = 5.6 Hz, J_{AX} = 8.9 Hz, $J_{AA'/XX'}$ = 2.5 Hz).

4-(Benzo[*d*][1,3]dioxol-5-yl)-6-(4-fluorobenzyl)-3-thioxo-3,4-dihydro-1,2,4-triazin-5(*2H*)-one (**9**). Azalactone derivative **5** (456 mg, 1.70 mmol) was heated under reflux in aqueous potassium hydroxide solution (1M, 5.1 mL) for 4 h. The reaction mixture was cooled, acidified with acetic acid and then thiosemicarbazide **8** (360 mg, 1.70 mmol) in EtOH (5.1 mL) was added. The reaction mixture was stirred under reflux for further 5 h. Then the mixture was diluted with water, extracted with ethyl acetate, and the organic phase was washed with brine, dried over Na_2SO_4, and concentrated under reduced pressure. The obtained crude product was purified by column chromatography, eluting

with *n*-hexane/EtOAc 85:15, to give the target compound **9** (377 mg, 62 % yield) as a yellow solid. ^{1}H-NMR ($CDCl_3$) δ ppm: 3.93 (s, 2H), 6.05 (d, 2H, *J* = 5.5 Hz), 6.60-6.69 (m, 2H), 6.91 (dd, 1H, *J* = 7.6, 1.0 Hz), 7.01 (double AA'XX', 2H, $^3J_{HF-o}$ = 9.5 Hz, J_{AX} = 8.7 Hz, $J_{AA'/XX'}$ = 2.5 Hz), 7.27-7.34 (m, 2H), 10.35 (bs, 1H).

4-(Benzo[*d*][1,3]dioxol-5-yl)-6-(4-fluorobenzyl)-3-(methylthio)-1,2,4-triazin-5(4*H*)-one (**10**). Compound **9** (659 mg, 1.84 mmol) dissolved in MeOH (1.8 mL) was reacted with methanolic sodium methoxide solution (25%, 0.44 mL) at 0 °C, then methyl iodide (0.15 mL, 1.8 mmol) was added and the reaction was stirred for 15 min. at rt. When the starting material was completely consumed (TLC), the solvent was evaporated to furnish intermediate **10** as a yellow solid (676 mg) that was used in the next step without further purification or NMR analysis.

4-Amino-3-(benzo[*d*][1,3]dioxol-5-ylamino)-6-(4-fluorobenzyl)-1,2,4-triazin-5(4*H*)-one (**11**). Hydrazine hydrate (50-60%, 1.3 mL) was added to a solution of intermediate **10** (683 mg, 1.84 mmol) in *i*PrOH (6.5 mL) and heated under reflux for 3 h. The solvent was removed under reduced pressure to obtain a residue that was purified by flash chromatography (eluent: *n*-hexane/EtOAc 8:2) to afford the title compound **11** (151 mg, 23 % yield). ^{1}H-NMR ($CDCl_3$) δ ppm: 4.05 (s, 2H), 4.67 (bs, 2H), 5.97 (s, 2H), 6.77 (d, 1H, *J* = 8.3 Hz), 6.91 (dd, 1H, *J* = 8.3, 2.2 Hz), 6.96 (double AA'XX', 2H, $^3J_{HF-o}$ = 9.5 Hz, J_{AX} = 8.7 Hz, $J_{AA'/XX'}$ = 2.5 Hz), 7.30 (d, 1H, *J* = 2.1 Hz), 7.35 (double AA'XX', 2H, $^4J_{HF-m}$ = 5.5 Hz, J_{AX} = 8.8 Hz, $J_{AA'/XX'}$ = 2.6 Hz).

3-(Benzo[*d*][1,3]dioxol-5-ylamino)-6-(4-fluorobenzyl)-1,2,4-triazin-5(2*H*)-one (**1**). To a cold suspension of compound **11** (151 mg, 0.425 mmol) in EtOH (7.6 mL) and concentrated HCl (1.5 mL), cold aqueous 10% sodium nitrite solution (3.8 mL) was added. The reaction was stirred overnight at rt. After removal of the solvent under reduced pressure, the crude product was purified by flash chromatography over silica gel column using a $CHCl_3$/MeOH 95:5 mixture as the eluent to obtain final compound **1** as a light-yellow solid (14.3 mg, 10 % yield). ^{1}H-NMR (DMSO-d_6) δ ppm: 3.79 (s, 2H), 6.01 (s, 2H), 6.80 (dd, 1H, *J* = 8.3, 2.1 Hz), 6.87 (d, 1H, *J* = 8.3 Hz), 7.06-7.14 (m, 3H), 7.29 (double AA'XX', 2H, $^4J_{HF-m}$ = 5.6 Hz, J_{AX} = 8.7 Hz, $J_{AA'/XX'}$ = 2.5 Hz), 9.12 (bs, 1H), 12.02 (bs, 1H).

^{13}C-NMR (DMSO-d_6) δ ppm: 35.11, 101.20, 104.73, 108.05, 114.91 (d, 2C, *J* = 21.2 Hz), 115.58, 130.94 (d, 2C, *J* = 8.0 Hz), 131.24, 132.63 (d, *J* = 3.4 Hz), 143.99, 147.22, 148.53, 153.78, 161.11 (d, *J* = 271.4 Hz), 162.16. HPLC analysis: retention time = 9.625 min; peak area, 97% (254 nm).

References.

1. Erlenmeyer E. Ueber die Condensation der Hippursäure mit Phtalsäureanhydrid und mit Benzaldehyd. Ann Chim Pharm 1893; 275:1–8.
2. Plöchl J. Über einige Derivate der Benzoylimdozimtsäure. Ber 1884; 17:1616–24.

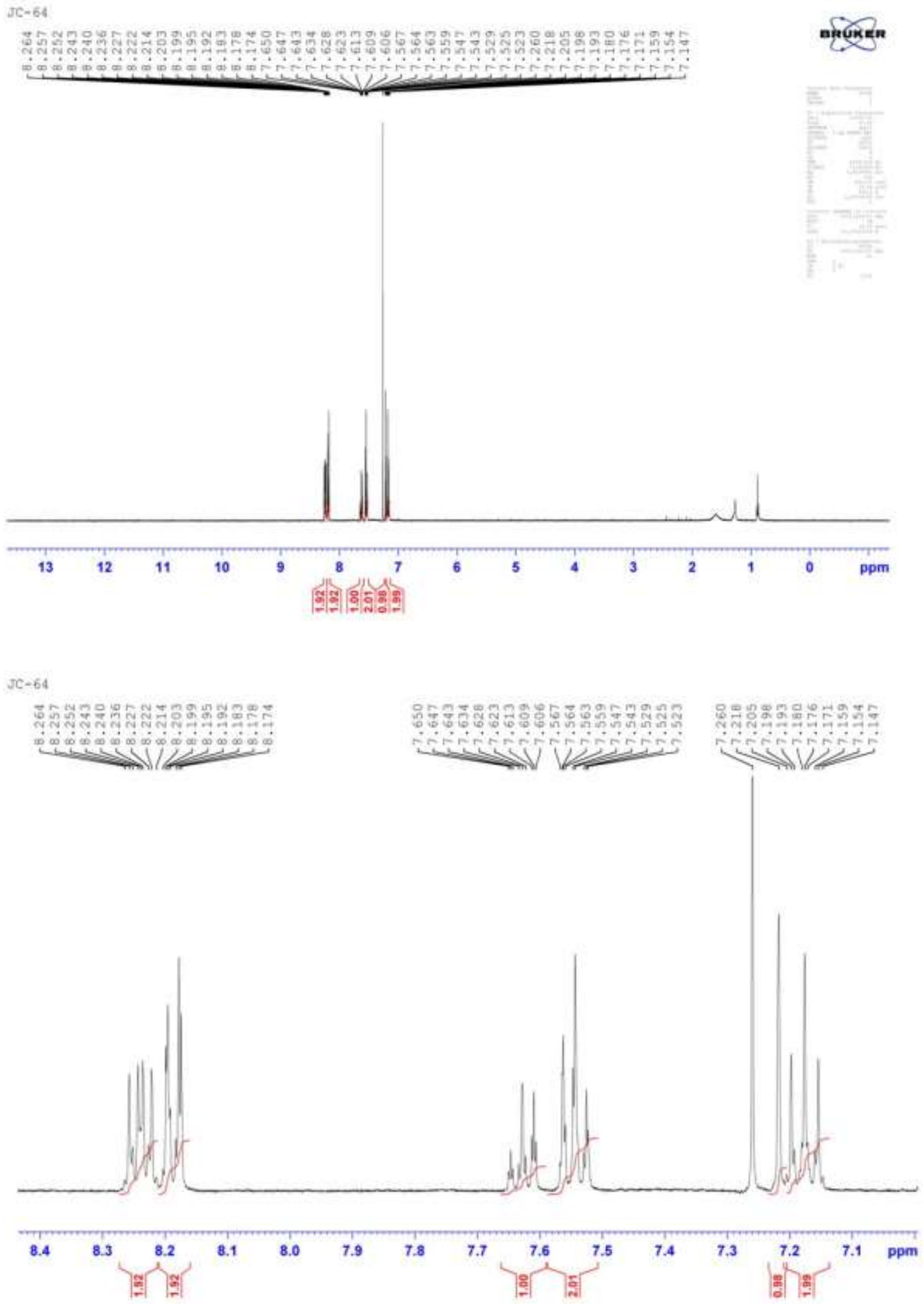

Figure S1. ^{1}H-NMR spectrum of intermediate **5**. Upper part: entire spectrum, lower part: expansion of aromatic protons.

==== Shimadzu LabSolutions Analysis Report ====

Sample Name : JC73_898uM
Sample ID : JC73_898uM
Data Filename : JC73_898uM_p.lcd
Method Filename : MAGL254.lcm

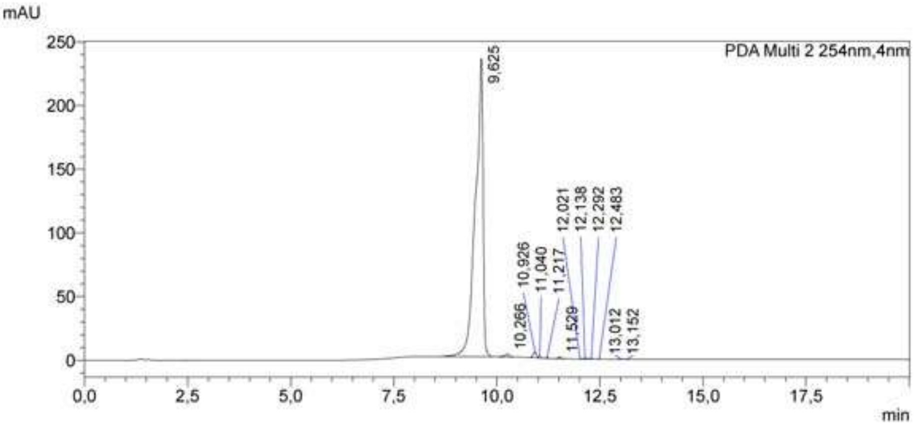

PDA Ch2 254nm

Peak#	Ret. Time	Area	Area%	Height
1	9,625	2990782	97,597	234570
2	10,266	19025	0,621	2375
3	10,926	26771	0,874	4953
4	11,040	5456	0,178	1322
5	11,217	2130	0,070	471
6	11,529	7817	0,255	1467
7	12,021	1034	0,034	272
8	12,138	3677	0,120	676
9	12,292	3493	0,114	496
10	12,483	1779	0,058	416
11	13,012	1413	0,046	260
12	13,152	1033	0,034	142
Total		3064409	100,000	247421

Figure S2. HPLC report of final compound **1**.

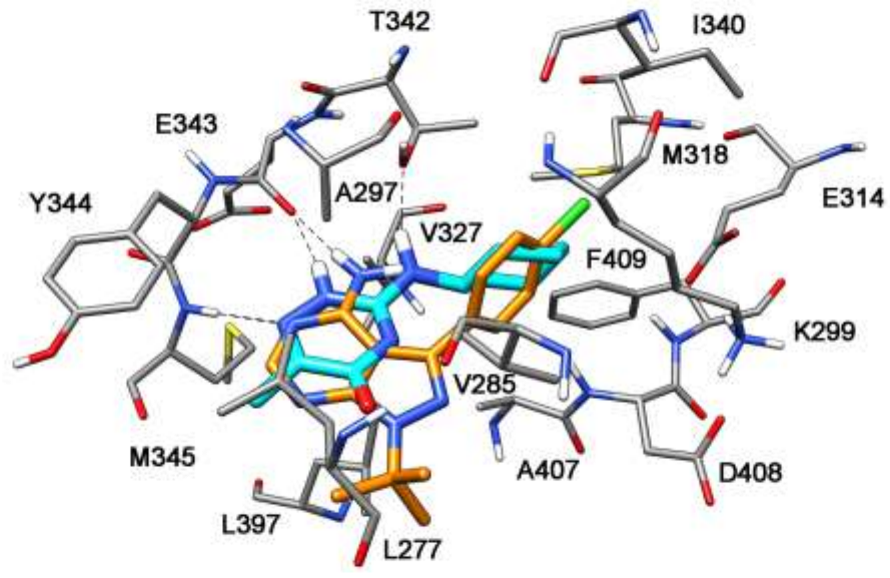

Figure S3. Superimposed structures of compound **3** (cyan) and the reference inhibitor PP2 (orange) in their predicted binding mode within Fyn catalytic site.
